# Nature-Inspired Gradient Material Structure with Exceptional Properties for Automotive Parts

**DOI:** 10.3390/ma18174069

**Published:** 2025-08-30

**Authors:** Xunchen Liu, Wenxuan Wang, Yingchao Zhao, Haibo Wu, Si Chen, Lanxin Wang

**Affiliations:** 1 School of Automotive Engineering, Wuhan University of Technology, Wuhan 430070, China; liuxunchen2021@163.com (X.L.); 358825@whut.edu.cn (W.W.); 2 Shandong Iron and Steel Group Rizhao Co., Ltd., Rizhao 276805, China; 13031670256@163.com (Y.Z.); jinan7665@163.com (H.W.); 3 School of Art and Design, Wuhan Institute of Technology, Wuhan 430070, China; 13227562786@163.com

**Keywords:** additive manufacturing, gradient structure, strengthening mechanisms, bioinspired design

## Abstract

Inspired by natural gradient structures observed in biological systems such as lobster exoskeletons and bamboo, this study proposes a biomimetic strategy for developing advanced automotive materials that achieve an optimal balance between strength and ductility. Against this backdrop, the present work systematically reviews the design principles underlying natural gradient structures and examines the advantages and limitations of current additive manufacturing—specifically selective laser melting (AM-SLM)—as well as conventional forming and machining processes, in fabricating nature-inspired architectures. The research systematically explores hierarchical gradient designs which endow materials with superior mechanical properties, including enhanced strength, stiffness, and energy absorption capabilities. Two primary strengthening mechanisms—hetero-deformation-induced (HDI) hardening and precipitation hardening—were employed to overcome the conventional strength–ductility trade-off. Gradient-structured materials were fabricated using selective laser melting, and microstructural analyses demonstrated that controlled interface zones and tailored precipitation distribution critically influence property improvements. Based on these findings, an integrated material design strategy combining nature-inspired gradient architectures with post-processing treatments is presented, providing a versatile methodology to meet the specific performance requirements of automotive components. Overall, this work offers new insights for developing next-generation lightweight structural materials with exceptional ductility and damage tolerance and establishes a framework for translating bioinspired concepts into practical engineering solutions.

## 1. Introduction

Metallic alloys remain the bedrock of load-bearing structures in aviation, aerospace, and—most prominently—the automotive industry. During the past two decades, steadily tightening demands on safety, emissions, and fuel economy have compelled automakers to seek body-in-white and chassis materials that marry high specific strength with outstanding ductility and superior energy absorption capability. First-generation advanced high-strength steels (AHSS-I), though inexpensive, can no longer satisfy aggressive lightweighting targets [1,2]. Second-generation transformation-induced plasticity steels (AHSS-II) deliver better performance but are hampered by intricate processing routes and costly alloying, preventing large-scale deployment [3,4,5]. Third-generation AHSS (AHSS-III) remains largely confined to laboratory validation and small-batch trials [6,7]. Alternatives such as aluminum, magnesium, and fiber-reinforced polymer composites offer density advantages, yet each falls short in crashworthiness, heat resistance, or recyclability, and thus cannot fully supplant steel. Overcoming the long-standing strength–ductility–toughness trade-off therefore stands as the central scientific and engineering challenge in developing the next generation of automotive structural materials.

From a materials and structural perspective, numerous natural systems feature specialized architectures that integrate relatively weak constituents to achieve mechanical robustness while retaining adequate ductility in biological organisms. The most classical structure is the gradient structure in exoskeleton of Homarus Americanus lobster [8] which brings a different stiffness and hardness. Another typical case is the gradient structure inside the cross-section of the Moso bamboo [9,10,11]. From the internal to external surface, the density and the size of the vessels are gradually decreased which accomplishes the fascinating performance. Thus, bamboo serves as an outstanding construction and tool material due to its high specific strength and toughness compared to many conventional materials.

Moreover, some methods can be utilized to reinforce the produced materials without sacrificing the ductility too much, including hetero-deformation-induced hardening, solid solution hardening, and precipitation hardening, etc. These methods were verified and mathematically analyzed by numerous works, and the underlying mechanisms were studied by microstructure investigation [12,13,14,15,16,17].

Meanwhile, additive manufacturing (AM)—in particular, selective laser melting (SLM)—has ushered in unprecedented processing freedom for the construction of multiscale, multi-material gradient architectures through on-demand design, layer-by-layer fabrication, and precise thermal management [18,19,20]. SLM enables rapid and tailored modulation of composition and microstructure gradients with micrometer-level spatial resolution by adjusting energy input profiles. In conjunction with targeted post-processing, it is possible to induce precipitation of L1_2_ or κ-carbide phases in designated regions, thus coupling HDI hardening with precipitation strengthening and creating novel automotive energy-absorbing units with exceptional damage tolerance.

Against this backdrop, the present work systematically reviews the design principles underlying natural gradient structures, such as hierarchical organization, functional grading, and interface optimization commonly found in biological materials. By understanding how nature achieves synergistic property combinations through spatially varied composition and structure, building on this analysis, we propose an integrated materials design strategy that synergistically combines hierarchical gradient structuring, HDI hardening, and precipitation hardening. The concept is exemplified through the use of a three-powder SLM system comprising 18Ni300, 316L, and CuSn10, and its applicability is demonstrated for key automotive components such as crash beams and battery trays [21,22,23]. Beyond pushing the strength–ductility frontier at the laboratory scale, this research is oriented toward cost-effectiveness, scalability, and recyclability, thereby laying both the theoretical and technological foundation for the next generation of lightweight automotive materials targeting new energy and intelligent vehicles by 2030.

## 2. Gradient Structure in Biological System

### 2.1. Design Principles of Biological Gradient Materials in Animals

Gradient means that materials are non-uniform, which usually appear near the surfaces or between two different materials [24]. Spatial gradients could be used to improve the mechanical and functional performance of materials, for example, to enhance the hardness and stiffness of the material. Nature gradient materials provide a wide range of inspiration for modern engineering materials, by using the properties’ gradients and heterogeneities of the materials [25,26]. With the evolution in nature, creatures use the basic components to assemble in ingenious ways to achieve better performance than synthetic materials.

According to chemical, structure, and geometrical variables to create gradients, gradients are fundamentally related to two sorts of ingredients, chemical compositions and structural characteristics.

#### 2.1.1. Chemical Compositions

Varying degrees with changes in chemical composition influence most gradients in biological materials. By changing the concentration and type of some chemical compositions such as bio-minerals, inorganic irons and biomolecules, and also the hydration degree, the properties of materials can be improved.

**Figure 1:**
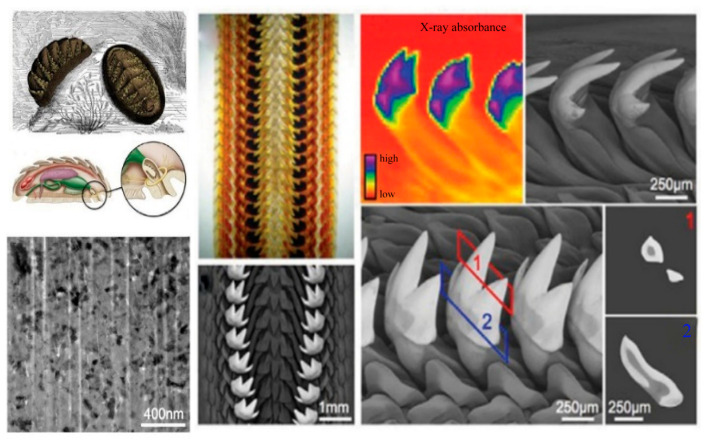
Morphological features of the chiton radula and synchrotron X-ray transmission map [27].

Mineralized biological materials achieve chemical gradients by regulating the degree of mineralization. As shown in Figure 1, chiton teeth exhibit exceptional hardness and stiffness by controlling the spatial assembly of organic and inorganic phases at the nanoscale. The figure displays both the morphological features of the chiton radula and colored maps derived from synchrotron X-ray transmission studies. These colored maps provide a visual representation of the electron density distribution within the chiton tooth structure, revealing regions of varying mineral content. Such imaging demonstrates how gradients in inorganic ions are spatially distributed, optimizing mechanical function through hierarchical mineralization.

**Figure 2:**
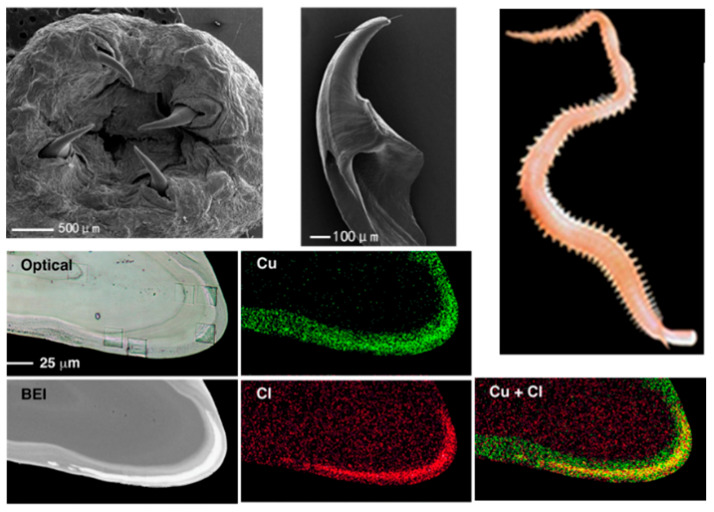
The jaws of bloodworm Glycera dibranchiate [29].

Figure 2 presents several views of the jaws of bloodworm Glycera dibranchiate, including scanning electron microscopy (SEM) and optical images, with the distance scales indicated as 500 μm and 100 μm for the SEM images, and 25 μm for the optical and elemental mapping images. The elemental distribution maps for copper (Cu) and chlorine (Cl) reveal a spatial gradient, with the overlay (Cu + Cl) highlighting areas of co-localization within the jaw tip. This multiscale imaging demonstrates how Cu ions are incorporated into the jaw structure, forming coordination complexes that result in a highly cross-linked molecular network, thus enhancing its mechanical strength and durability [28].

#### 2.1.2. Structural Characteristics

The structure of the bio-materials also has a great effect on improving and enhancing the properties of the materials. The generation of gradients is related to these aspects of characteristics of materials, i.e., the arrangement, distribution, dimension, orientation, and also the gradient structure which appear between two different materials [30].

**Figure 3:**
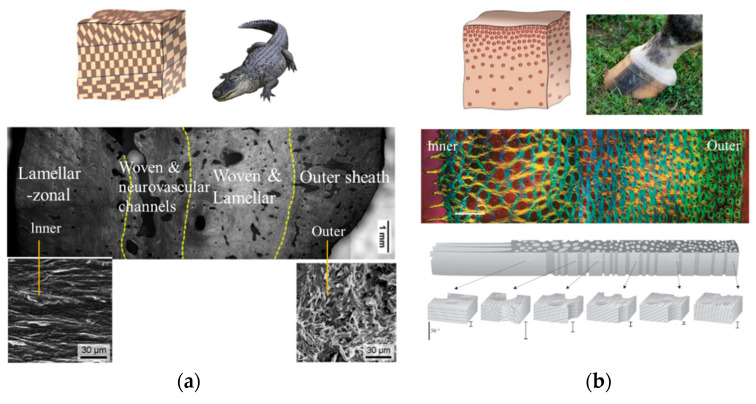
(**a**) Structure of alligator osteoderms [24,31]. (**b**) Structure of horse hoof [24,32].

Figure 3a presents the hierarchical structure of alligator osteoderms, showing a transition from hard woven bone in the dorsal (outer) region to softer lamellar-zonal bone in the abdominal (inner) region. The sectional image marks four distinct regions: lamellar-zonal, woven and neurovascular channels, woven and lamellar, and outer sheath, with corresponding SEM micrographs at a 30 μm scale further illustrating the inner (lamellar-zonal) and outer (outer sheath) microstructures. This gradient, multi-layered architecture enhances the osteoderms’ overall capacity for energy absorption, providing effective protection as well as flexibility for the alligator [31].

Figure 3b depicts the gradient tubular structure of a horse hoof. The colored cross-sectional image and schematic show a gradual change in tubule orientation and density from the outer surface to the inner regions. This non-uniform organizational gradient results in varying mechanical properties across different regions of the hoof, optimizing its ability to absorb and dissipate impact forces from the ground and thereby supporting the critical functional needs of the hoof [32].

**Figure 4:**
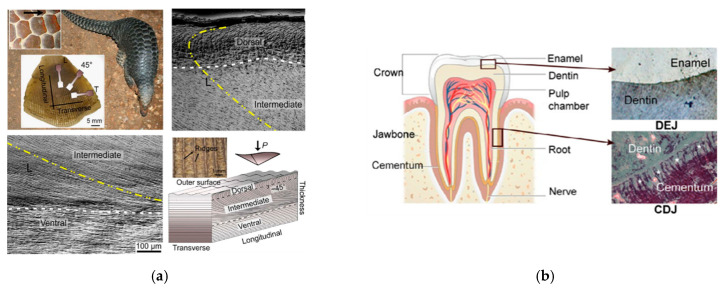
(**a**) Structure of pangolin scale [33]. (**b**) Structure of human tooth [24,34].

The dimensions and spatial arrangement of structural units play a critical role in determining the performance and functionality of biological materials. For instance, modulating the dimensions of material components—such as the exoskeleton of Homarus Americanus—can facilitate the development of gradient structures, thereby enhancing mechanical properties [8]. Similarly, altering the orientation of structural units provides an additional strategy for introducing functional gradients. Figure 4a depicts the hierarchical architecture of a pangolin scale, with a focus on the gradual reorientation of structural units in the intermediate region. The white line indicates the orientation from the interior to the exterior, whereas the yellow line represents an approximately 45-degree deviation; compared with the white-line orientation, the yellow-line region exhibits increased surface hardness and stiffness, significantly enhancing impact resistance and protective capability [33]. Figure 4b illustrates the gradient interface between human enamel and dentin within a tooth [24]. This transition zone, characterized by a gradual shift from highly mineralized enamel to tough dentin, enables efficient load distribution during mastication. Such a design not only optimizes mechanical performance but also ensures robust attachment and load-bearing capacity of the tooth.

#### 2.1.3. Exoskeleton of Homarus Americanus Lobster

Exoskeleton of the Homarus American lobster is a typical example that uses dimensions of structural gradients to improve the properties of the exoskeleton. Exoskeleton of Homarus Americanus lobster is composed of mineralized fibers and chitin-based tissue with three main layers, which is a strictly hierarchical organization from the nano-level to the macro-level. The three main layers are epicuticle, exocuticle, and endocuticle; exocuticle and endocuticle layers mainly carry the force and load, which are composed of chitin-based tissue. The twisted plywood is formed by a certain sequence of the chitin–protein layers, which is the characteristic part of the structure.

**Figure 5:**
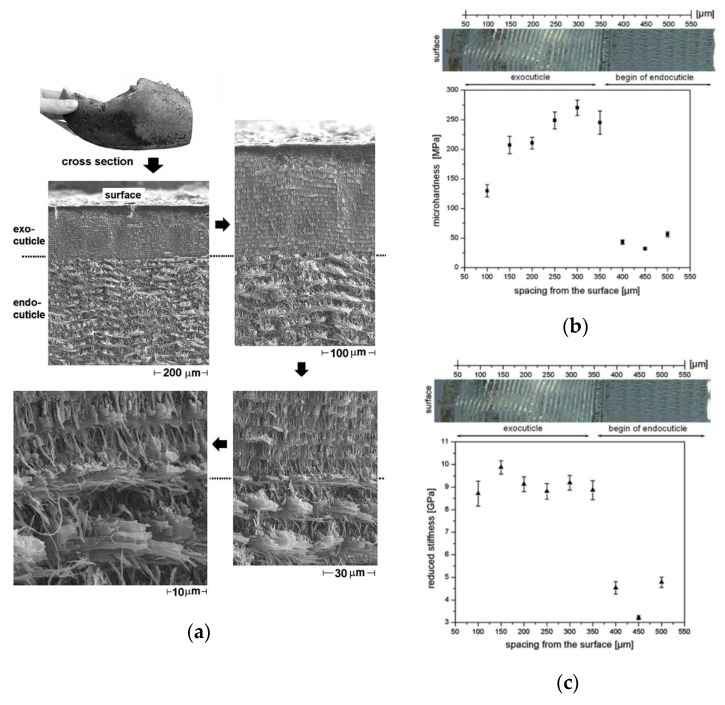
(**a**) Micrographs showing exocuticle (**b**) Hardness distribution across layers (**c**) Reduced stiffness distribution Structure of exoskeleton of Homarus Americanus lobster and the hardness and the reduced stiffness [8].

Figure 5a shows the micrographs of the claw of the lobster, especially showing the obvious change between the exocuticle and the endocuticle. Twisted plywood structure changed a lot between the exocuticle and the endocuticle, and the density of chitin–protein fibers becomes finer from the outer layer to the inner layers. The thickness of the twisted plywood layers also increases from the outer surface to the inner, which is similar to the Hall–Petch relationship. Hall–Petch relation explains the relation between the dimension of the crystal and the strength of the materials. For the same volume of metal, the fine crystal has a relatively large surface area and can withstand a large strength. This requires a larger force to make the crystal slip. The smaller the grain size, the larger the yield limit of the material.

By analogizing the Hall–Petch relation, the gradient of chitin–protein fibers’ dimensions makes the structure harder and stiffer. The finer the structure is, the higher its hardness and stiffness are. The structure results in graded properties across the tissue as shown in Figure 5b,c. The hardness gradually increases with the gradients within the exocuticle and the endocuticle layers, and there is an abrupt change between these two layers.

Exoskeleton of Homarus Americanus lobster offers a viable means for the development of gradients in these materials through alterations in the dimensions of structural units, which could be applied in engineering synthetic materials to improve mechanical properties of materials.

### 2.2. Design Principles of Biological Gradient Materials Inplants

Plants have been wildly used in many fields in our daily life, and many of them have fascinating inner structures and mechanisms. Bamboo, as a well-known special-looking plant, has a long history being used as building and tool materials. According to the FAO’s 2010 assessment, the total 31.4 million hectares of bamboo are more concentrated in China, India, and Brazil [35].

**Figure 6:**
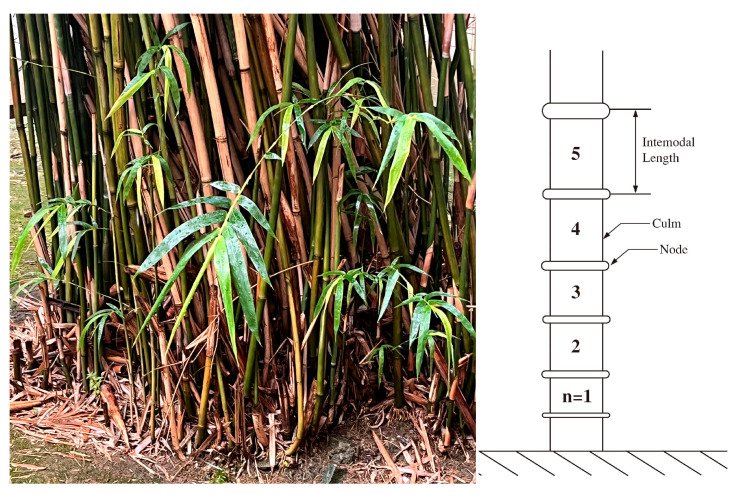
Moso bamboo and schematic diagram.

Bamboo is a perennial member of the family Poaceae and is one of the fastest-growing plants in the world a. Its main structure is hollow cylindrical tube, also known as a culm, which is divided into multiple sections by tangential nodes; the regions between two nodes are called internodes. Generally, both the diameter of the bamboo’s circular cross-section and its wall thickness decrease with increasing height [10]. As shown in Figure 6, Moso bamboo exhibits a hierarchical structure, which plays a crucial role in achieving its outstanding mechanical properties.

#### 2.2.1. Moso Bamboo’s Gradient Distribution

Moso bamboo, as the most commercially cultivated species of its kind in China, has an unusual property in its inner structure that raised a great interest among biological and mechanical scientists.

**Figure 7:**
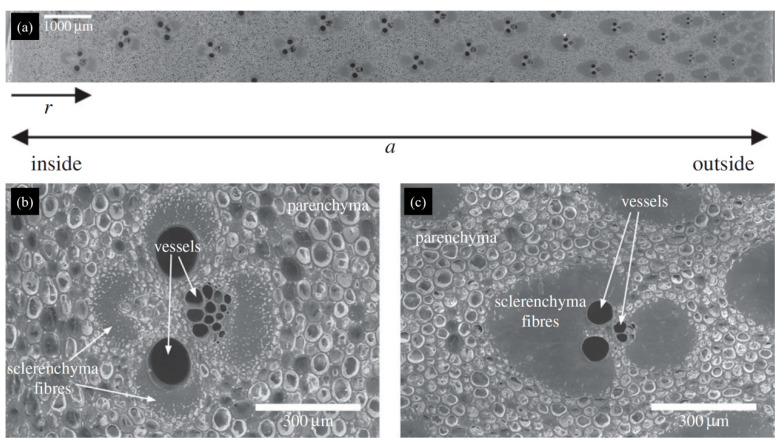
(**a**) Inner distribution of the bamboo’s wall. (**b**) SEM micrograph of inner wall specimen. (**c**) SEM micrograph of outer wall specimen [9].

The wall of Moso bamboo exhibits a distinct gradient distribution in its microstructure. As shown in Figure 7a, the inner region of the bamboo wall contains a much greater number of vessels, represented by the dark groups of dots. These vessel-rich areas are interspersed with sclerenchyma fibers—appearing as faint gray zones—and filled in by parenchyma tissue, which together result in a relatively loose texture.

Further insights into this organization can be gained from the SEM micrograph of the inner wall specimen, as depicted in Figure 7b. Here, the vessels and fibers are arranged in a less compact manner, with a larger fraction of vessel regions and more apparent gaps. This gradient in microstructural density continues toward the outer wall, where the tissue transitions into densely packed sclerenchyma fibers and features fewer vessels. Consequently, the outer portion of the bamboo wall possesses much greater toughness and density, supporting its long-standing application in high-strength structures and historical weapons.

#### 2.2.2. Mechanical Tests and Microstructure Investigation on Bamboo Wall

**Figure 8:**
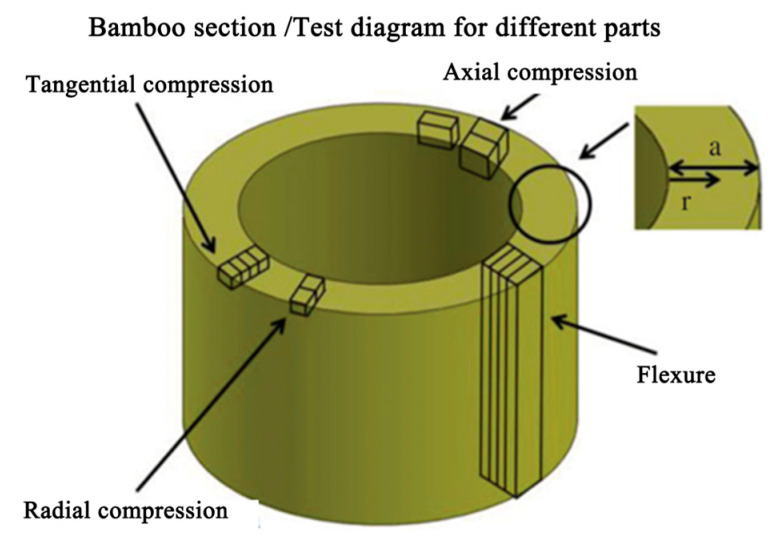
Sampling positions of the bamboo section and test methods [9].

The bamboo itself is strong enough to sustain the powerful wind and work as a trusty supporting material, while remaining quite light. Scientists have performed many tests about bamboo’s section, as illustrated in Figure 8, including tangential compression, radial compression, flexure, and axial compression, as well as the overall test over the entire section.

**Figure 9:**
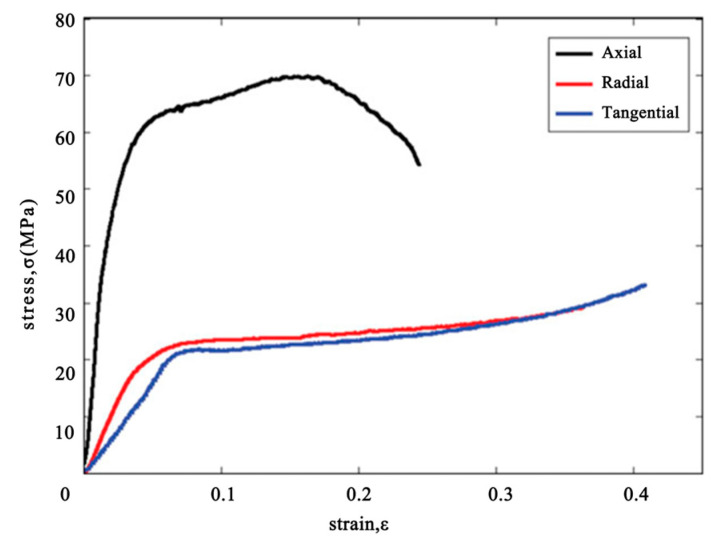
Overall stress–strain curve with a whole section of the bamboo [9].

As shown in Figure 9 above, the bamboo section has an outstanding performance in the axial stress test, naturally, compared to the other directions. The presence of a hollow interior in the bamboo cross-section leads to a considerable reduction in structural mass, while allowing the material to retain superior toughness and mechanical stability compared to solid counterparts of similar dimensions.

**Figure 10:**
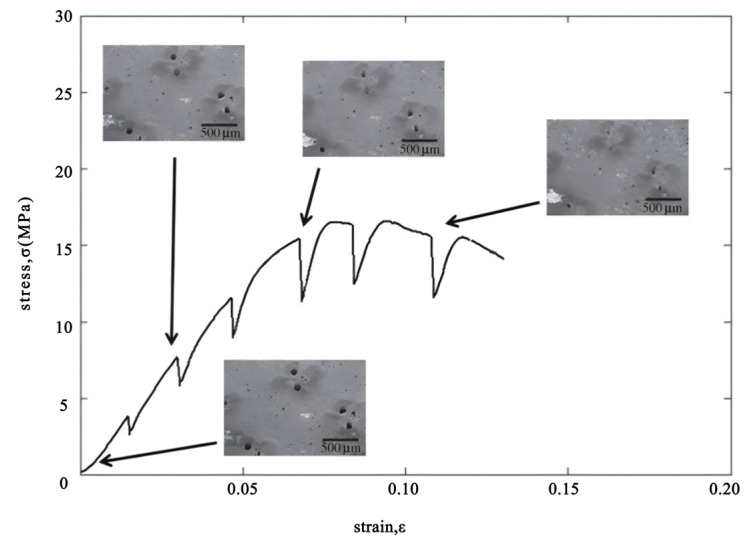
The stress–strain curve with radial compression [9].

Interestingly, the results of axial compression tests on the bamboo wall specimens revealed behavior that deviates from those expected in conventional materials. As shown in Figure 10, the stress–strain curve obtained under radial compression displays a series of troughs, rather than following the typical smooth profile. This pattern indicates that, during deformation, the specimen experiences multiple stages of resistance and localized failure, reflecting the complex hierarchical structure and energy-dissipating mechanisms inherent in bamboo.

**Figure 11:**
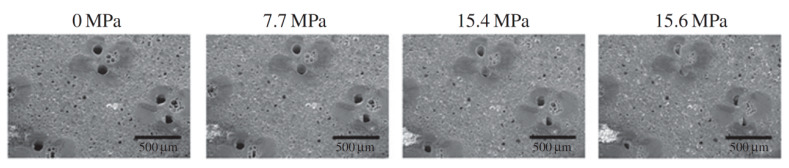
Detailed SEM micrographs of the same position in different pressures [9].

As observed in the SEM images presented in Figure 11, the vessels in the bamboo specimen gradually collapse as the test progresses. A closer examination reveals that the parenchyma tissue outside the vessels undergoes compression, while the surrounding sclerenchyma fibers largely remain unchanged. This difference in deformation may contribute to the initial crushing of the vessels [10,11].

The results of this test indicate that energy absorption occurs at each stage of structural change during compression. Although the vessels collapse throughout the process, the overall toughness of the specimen is retained, suggesting significant potential for energy absorption in accidental situations. Materials exhibiting such structural characteristics may demonstrate high axial toughness combined with notable radial energy-absorbing capacity, making them promising candidates for use in structural components such as automobile support beams.

So, overall, the unique inner structure of bamboo, with a significant radial gradient in the wall, provides high compressive strength in the axial direction. In addition, the combination of vessels, sclerenchyma fibers, and parenchyma cells produces an efficient radial energy absorption system while maintaining mechanical stability. Due to these properties, bamboo achieves considerable weight reduction while its primary structures remain mechanically robust through optimized mineral distribution.

Inspired by the sophisticated gradient structures found in biological systems, we translated these natural design principles into a targeted strategy for advanced engineering materials. Each biological case discussed above offers explicit guidance for parameter selection in synthetic gradient materials: the squid beak’s gradation in stiffness with depth reveals the value of controlling macromolecular architecture and hydration across spatial domains; bamboo’s hierarchical arrangement of fiber bundles demonstrates how tuning the spatial distribution of reinforcement phases can optimize both strength and ductility; and the spiral mineralization in lobster exoskeletons directly informs the design of layerwise heterogeneity in additive manufacturing processes. By systematically analyzing these natural prototypes, we identified critical variables—including compositional gradients, fiber orientation, and phase dispersion—that now underpin our materials design framework. In the following section, we elaborate how these bioinspired parameters are integrated into our proposed materials strategy, establishing a direct connection between natural model systems and engineering implementation.

## 3. Current Manufacturing Processes

Moreover, by integrating the bioinspired design principles discussed in the previous section, AM provides an effective pathway to realize complex gradient architectures found in nature within engineered materials [36]. The layer-by-layer deposition inherent to AM processes matches the hierarchical and spatially heterogeneous features observed in squid beaks, bamboo, and lobster exoskeletons, enabling precise control over compositional and structural gradients at multiple scales [37]. This synergy between biological principles and advanced manufacturing not only enhances the mechanical performance of lightweight structural components but also accelerates the practical application of bioinspired strategies in automotive and other high-performance engineering domains. Thus, the proposed materials strategy is inherently compatible with the latest advances in digital manufacturing, allowing for flexible, efficient, and scalable production of next-generation gradient materials [38].

**Figure 12:**
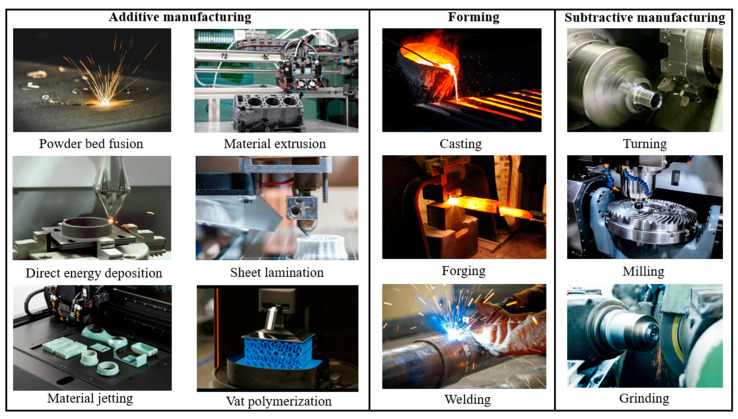
Type of manufacturing technology [39].

As shown in Figure 12, the manufacturing can be divided into additive manufacturing, subtractive manufacturing, and forming.

Additive manufacturing is combined with computer-aided design, materials processing and forming technology. Based on digital model files and numerical control systems by the software, special metallic materials, non-metallic materials, and medical biological materials are used to manufacture the parts. The manufacturing process is layer-by-layer deposition by extrusion, sintering, melting, curing light, and injection. Three elements of the AM are digital manufacturing, layer-by-layer, and materials deposition [39]. Powder bed fusion, direct energy deposition, material jetting, material extrusion, sheet lamination, binder jetting, and vat polymerization are the common forms of additive manufacturing.

Particularly, the AM technology needs less machining, less assembly, and fewer manufacturing steps, which saves large energy consumption. It illustrates that the AM technology is a sustainable and satisfied manufacturing technology for the light-weight components on the automotive. Because it can achieve the preparation of components with complex structures, additive manufacturing technology has advantages in the fabrication of parts with nature-inspired material structures.

## 4. Material Strengthening Methodology

### 4.1. Hetero-Deformation-Induced Hardening

**Figure 13:**
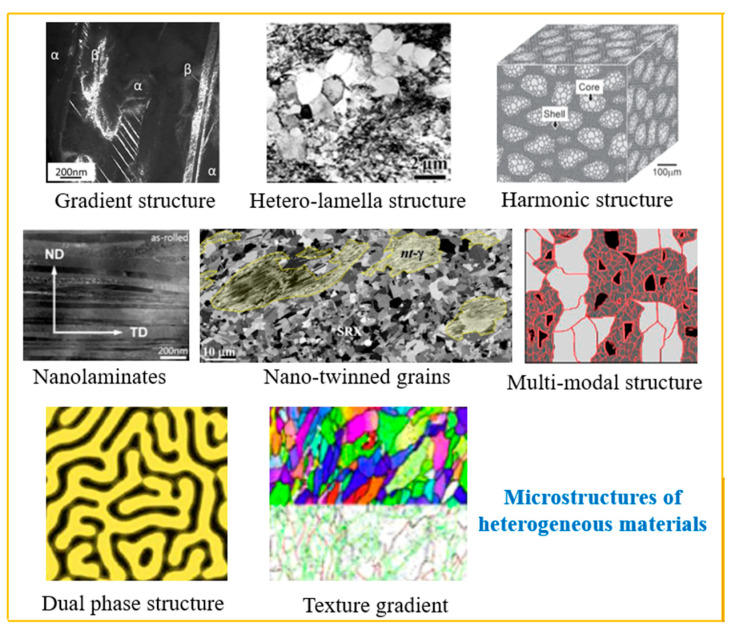
Materials microstructure classification [13].

According to the distribution uniformity, materials can be divided into homogeneous materials and heterogeneous materials. As shown in the following Figure 13, the heterogeneous materials include gradient structure, hetero-lamella structure, harmonic structure, nanolaminates, nano-twinned grains, multi-modal structure, dual-phase structure, texture gradient structure, etc. [12,13]. Usually, the mechanical property of the heterogeneous materials is better.

**Figure 14:**
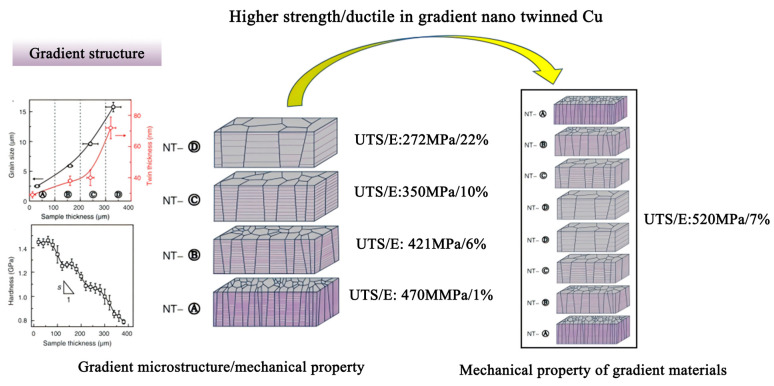
Higher strength/ductile in gradient nano-twinned Cu [40].

Figure 14 shows the design of gradient nano-twinned Cu. From layer A to layer D, the grain size tends to gradually increase, and the hardness, characterized by Vickers hardness, gradually decreases. UTS/E values for each layer are 470 MPa/1%, 421 MPa/6%, 350 MPa/10%, and 272 MPa/22%, respectively. The overall UTS/E of the gradient nano-twinned Cu is 520 MPa/7%, which demonstrates the effective strength–ductility trade-off achieved by the gradient structure [40]. The correlation between grain size, twin thickness, and Vickers hardness reveals that by engineering layered gradients in microstructure, it is possible to simultaneously achieve high strength and improved ductility. This highlights the potential of gradient architectures in optimizing the mechanical properties of advanced structural materials.

**Figure 15:**
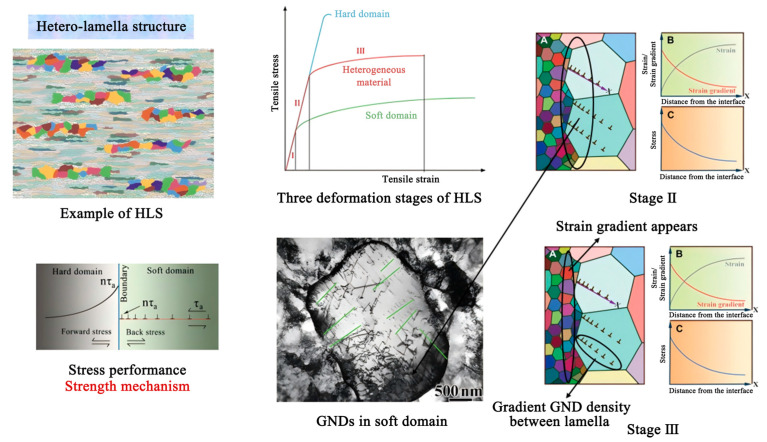
Hetero-lamella structure and strengthening mechanism [42,43,44,45,46].

In the hetero-lamella structure, the gradient also exists on the interface between hard domain and soft domain. The hetero-lamella structure is shown in Figure 15, the small grains belong to the hard domain, and the large grains belong to the soft domain. Three stages take place in the deformation [12,13,41]:

1. At first stage, external stress is small. Elastic deformations exist in the hard and soft domains.
2. As shown in Figure 15, At second stage, as the external stress increases, soft grains begin to initiate plastic deformation through dislocation slip, while the hard grains remain elastically deformed. Plastic incompatibility occurs at the heterointerface, which leads to accumulation of interfacial geometrically necessary dislocations (GNDs) causing strain gradients (in the soft domain). Accumulation of the GNDs creates long-range back-stress (internal stress, opposite to the external stress) in soft domain. At the same time, the back-stress induces forward-stress (internal stress, same direction as the external stress) in the hard domain. Particularly, the back-stress and the forward-stress act as a pair of co-existing stresses in the heterointerface; they are the same in magnitude, but in opposite direction.
  In the case of increasing external stress, ductility of soft domain is enhanced through continuous accumulation of the GNDs, and the back-stress generated by the accumulation of the GNDs also improves strength of soft domain.
  In the hard domain, direction of the forward-stress is the same as the external stress, and the forward-stress induces deformation and “softening” of the hard domain (under the action of a certain external stress, the forward-stress makes the hard domain deform more and improves ductility of the hard domain). However, in this stage, the external stress is limited, accumulation of interfacial GNDs is limited, the back-stress is limited, and the forward-stress is limited. The forward-stress and the external stress are not enough to make the hard domain deform. So, the effect of the forward-stress in the hard domain is only a tendency (the forward-stress makes the hard domain near the interfaces easier to yield).
3. As shown in Figure 15, At third stage, the external stress and the forward-stress are enough, plastic deformations exist in the hard and soft domains. Situation in the soft domain is similar to that in the second stage: in the case of the increasing external stress, ductility of the soft domain is enhanced through continuous accumulation of the GNDs, and the back-stress generated by the accumulation of the GNDs also improves the strength of the soft domain. Situation in the hard domain is different from in second stage: the forward-stress makes the hard domain deform more under certain external stress and improves the ductility of the hard domain.

Under combination action of the forward-stress and the back-stress, the hetero-lamella structure can display the exceptional strength and work-hardening ability.

**Figure 16:**
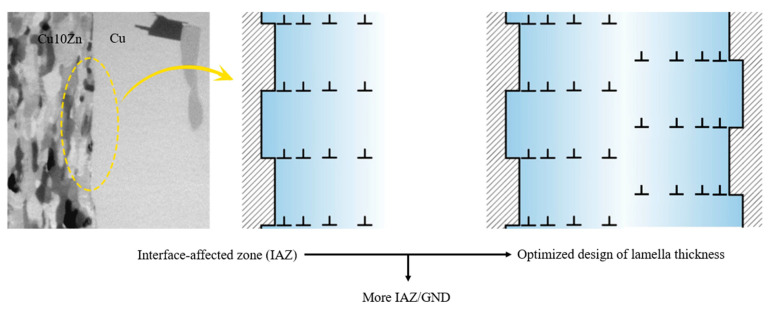
Further improvement of strength/ductile by IAZ [48].

According to the above strengthening mechanism, the GNDs induce the forward-stress and back-stress, which improve the mechanical property of hetero-lamella structure. Therefore, it is an interesting idea to calculate interface-affected zone (IAZ) and optimize lamella thickness, as shown in Figure 16 [47]. The lamella happens to consist of the IAZs; the IAZ is larger and the GNDs are more, which realizes further improvement of the strength and the ductile.

**Figure 17:**
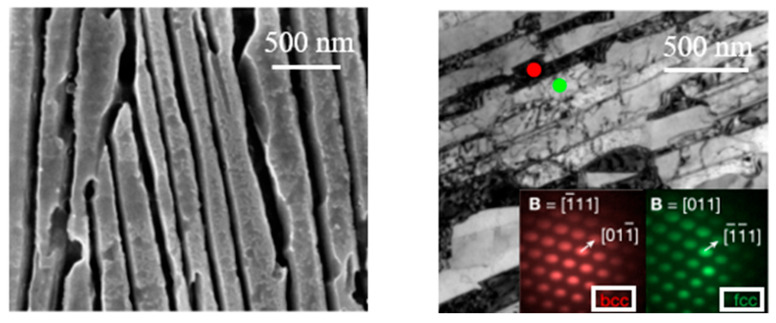
Nano lamellar high-entropy alloy microstructure [14].

In some nanolaminates, the interface plays an important role in improving the strength and the ductility of the material. Figure 17 shows a kind of nano lamellar high-entropy alloy (HEA) microstructure [14]. High-entropy alloys are known for their exceptional mechanical properties, such as high strength, good ductility, and excellent thermal stability, especially when synthesized with a nano lamellar structure [49]. The selection of this alloy in our work is significant because it demonstrates how a gradient or layered microstructure can synergistically enhance both strength and ductility, serving as a model system for advanced material design strategies.

**Figure 18:**
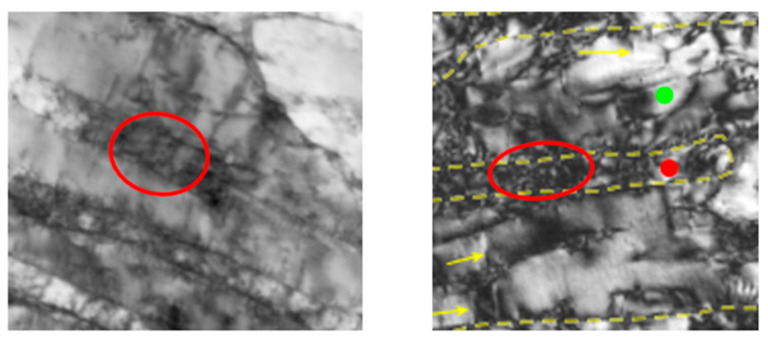
Precession electron diffraction [14].

At greater magnification, dislocations block, and dislocations store abilities are found in nano lamellar interfaces, as shown in Figure 18. These features contribute to enhanced strength and ductility, as highlighted in red in the image [41].

**Figure 19:**
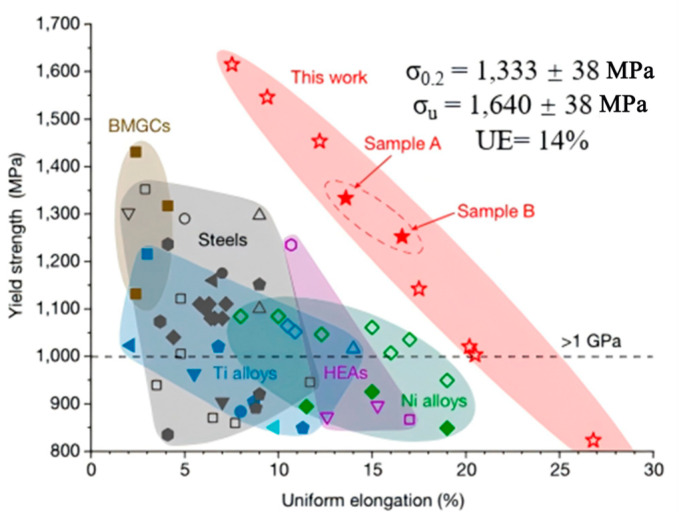
Exceptional combination of strength and ductility in natural structures [14]. (The red area represents EHEAs, the purple area represents HEAs, the green area represents Ni alloys, the blue area represents Ti alloys, the gray area represents Steels, and the brown area represents BMGCs.).

Samples A and B in Figure 19 correspond to AM eutectic EHEAs with distinctly tailored microstructures. Sample A is the as-printed state, displaying a remarkable 0.2%-offset yield strength of 1333 ± 38 MPa and a uniform elongation of ~14%. Sample B represents the alloy after optimized annealing treatment (such as 600 °C for 5 h), which further increases the yield strength and ultimate tensile strength to approximately 1.6 GPa and 1.9 GPa, respectively, with a uniform elongation of 7.5%.

Such impressive combinations of strength and ductility originate from the nanoscale lamellar architecture and the tunable interlamellar spacing characteristic of AM EHEAs. In this study, the reason for selecting this alloy lies in its unique capability for microstructural modulation through additive manufacturing and heat treatment, enabling the realization of nearly isotropic and superior mechanical properties. This approach demonstrates the promising strategy of leveraging hierarchical structural design and compositional complexity to optimize the mechanical performance of advanced alloys.

### 4.2. Hydrogen Embrittlement

Hydrogen embrittlement, in particular, can significantly impair the mechanical performance of metals and alloys, including those produced by additive manufacturing [50,51]. Due to hydrogen’s small atomic radius and high mobility, it readily diffuses into metallic lattices, accumulating at defects, interfaces, and grain boundaries. This accumulation initiates a series of microstructural changes that can drastically reduce ductility and lead to premature failure.

**Figure 20:**
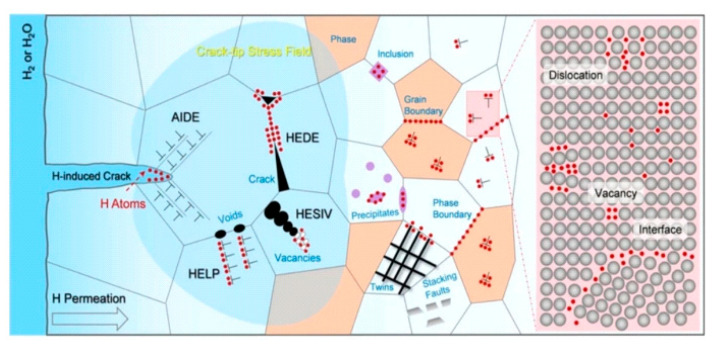
Active mechanisms of hydrogen embrittlement [50].

As illustrated in Figure 20, multiple mechanisms are involved in hydrogen-induced degradation—such as Hydrogen-Enhanced Decohesion (HEDE) [52], Hydrogen-Enhanced Localized Plasticity (HELP) [53], and Hydrogen-Enhanced Strain-Induced Vacancy (HESIV) [54]. The presence of hydrogen may also lead to the generation of vacancies, twins, stacking faults, and voids, all of which serve as stress concentrators and weaken the structural integrity of the part.

**Figure 21:**
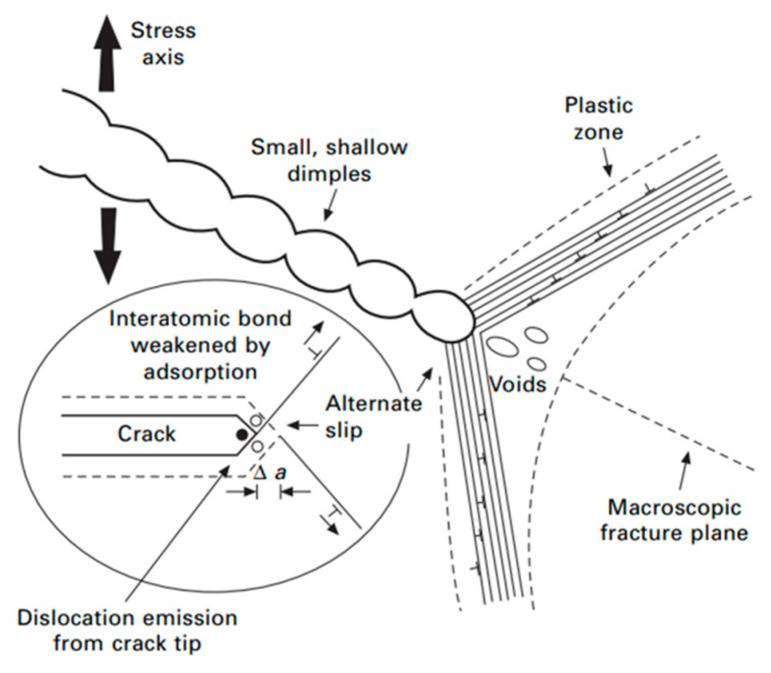
Schematic of adsorption-induced dislocation emission at a crack tip (AIDE) [56].

Another significant mechanism is adsorption-induced dislocation emission (AIDE), shown in Figure 21. The AIDE theory suggests that crack growth is facilitated not only by dislocation emission at the crack tip, but also by the nucleation and growth of microvoids ahead of the crack [55]. Hydrogen adsorption weakens interatomic bonds, requiring a lower stress for dislocation emission. Under sufficient stress, particularly in the presence of hydrogen diffusion and preexisting voids or cracks, both dislocation activity and alternating slip can be triggered, promoting crack propagation and macroscopic fracture. This highlights the complexity and danger of hydrogen embrittlement in environments relevant to additive manufacturing.

Property gradients and microstructural heterogeneity, intentionally introduced to achieve functional objectives in gradient materials, may further create preferential sites for hydrogen accumulation and embrittlement. As the property contrast rises—especially at interfaces—local stresses and hydrogen segregation are enhanced, making these regions more vulnerable to crack nucleation and propagation under service loads.

### 4.3. Precipitation Hardening

The development of engineering materials is always conducted towards high mechanical strength and excellent ductility as much as possible [15,57,58]. However, there is a trade-off between high mechanical strength and ductility. In order to overcome this issue, a series of novel materials and designs have been proposed to achieve this target. Wu et al. investigated the mechanical performance of $Al_{0.1}CoCrFeNi$ HEA with cold working and annealing post-processing to bring out the excellent properties with 711 MPa yield strength, 928 MPa ultimate tensile strength, and 30.3% uniform elongation [15]. Their amazing enhancement was attributed to the complex heterogenous structures by combining the non-recrystallized and recrystallized grains which induced the high-density dislocation around the grain boundaries and nano-twinning inside the non-recrystallized grains. Another team attained high mechanical performance (yield strength of 650 MPa, tensile strength of 853 MPa, and elongation of 34%) through doping the nitrogen into the FeCoNiCr HEA without heat treatment. Also, the grain sizes with large variations were observed in the microstructure of N-doped HEA, which formed numerous slip bands and piled-up geometrically necessary dislocation between the coarse grain and fine grain [58]. Moreover, the stacked faults (SFs) [15,16,57] and combination of body-centered cubic (BCC) and face-centered cubic (FCC) [16] can also improve the material properties.

Besides the mechanisms mentioned above, precipitation is also a promising way to enhance the strength [16,17,57]. The L1_2_ precipitates are ordered intermetallic phases with a face-centered cubic structure. They are distributed within the disordered FCC matrix of the alloy. Compared to the matrix, L1_2_ phases have long-range atomic order and effectively hinder dislocation motion, thus enhancing the strength of the material. Lu’s group has suggested a process to introduce the $L1_{2}$ precipitates into the $Ni_{46}Cr_{23}Fe_{23}Al_{4}Ti_{4}$ medium-entropy alloy (MEA) and set the $Ni_{50}Cr_{25}Fe_{25}$ MEA as contrast. These two alloys passed the cold rolling to achieve the 80% thickness reduction. Next, the $Ni_{46}Cr_{23}Fe_{23}Al_{4}Ti_{4}$ MEA was annealed at 1323 K for 15 min and then aged under 973 K for 8 h. The contrasting $Ni_{50}Cr_{25}Fe_{25}$ MEA was recrystallization-treated at 1473 K for 1 h. The results indicated a yield strength of approximately 1203 MPa, an ultimate tensile strength of about 1633 MPa, and an elongation of 28.7%, demonstrating a combination of high mechanical strength and ductility.

**Figure 22:**
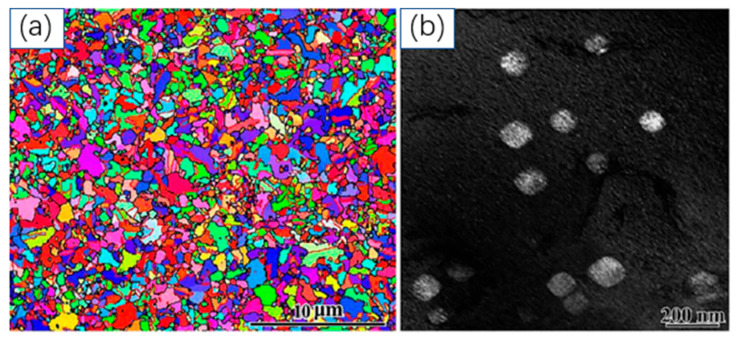
Microstructure investigation of $Ni_{46}Cr_{23}Fe_{23}Al_{4}Ti_{4}$ MEA after 1323 K-15 min annealing. (**a**) Inverse pole figure image revealing the different grain sizes. (**b**) Dark field image revealing the large $L1_{2}$ precipitation inside the FCC matrix [17].

**Figure 23:**
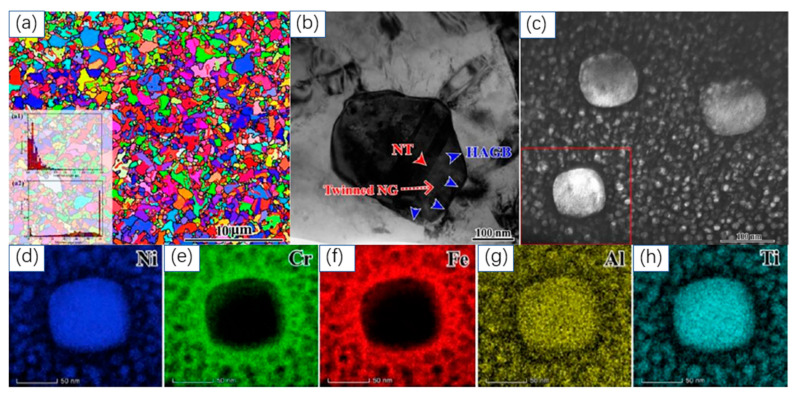
Microstructure investigation of $Ni_{46}Cr_{23}Fe_{23}Al_{4}Ti_{4}$ MEA after 973 K-8 h aging. (**a**): Inverse pole figure image; inset figure shows grain size distribution and misorientation after annealing and aging. (**b**): TEM bright field image showing twinned nano-grain. (**c**): Dark field image showing heterogeneous $L1_{2}$ precipitates. (**d**–**h**): EDS mapping indicating the $L1_{2}$ precipitates element composition of the red rectangle in (**c**) [17].

Figure 22 and Figure 23 present the detailed microstructure of the $Ni_{46}Cr_{23}Fe_{23}Al_{4}Ti_{4}$ MEA after annealing (1323 K for 15 min) and subsequent aging treatment (973 K for 8 h), respectively. As shown in Figure 22a, the EBSD-IPF map reveals a heterogeneous grain structure with sizes spanning from nanometers to several micrometers after annealing. This heterogeneity is statistically confirmed, contributing to the mechanical robustness of the alloy. Figure 22b further displays the random distribution of large L1_2_ precipitates with near-cubic shapes and rounded corners within the FCC matrix. Following aging, Figure 23a indicates that the three-level heterogeneous grain structure is preserved, with an average grain size of approximately 0.41 μm. The statistical inset shows that nano-grains (<250 nm) account for 46%, ultra-fine grains (250 nm–1 μm) for 49%, and micrometer-sized grains (>1 μm) for 5% of the total grain population. The presence of abundant annealing twins, as revealed by EBSD, suggests a low stacking fault energy, and these twins facilitate the formation and retention of the hierarchical grain structure. Figure 23b,c highlights the formation and distribution of twinned nano-grains at high-angle grain boundaries, a phenomenon common in low SFE metals. Additionally, Figure 23c demonstrates a high density of fine, nearly spherical L1_2_ precipitates formed during aging, with a few larger cubic precipitates remaining from the initial annealing process. This morphological evolution is mainly attributed to changes in lattice mismatch during heat treatment.

Figure 23d–h presents the elemental mapping of a representative fine L1_2_ precipitate, clearly revealing its chemical composition and partitioning behavior in the aged Al_4_Ti_4_ MEA. These mappings demonstrate that the precipitate is significantly enriched in Ni, Al, and Ti, while Fe and Cr are substantially depleted within the precipitate region, leading to a stable Ni-Al-Ti-rich L1_2_ phase embedded in an Fe-Cr-rich fcc matrix. This trend is quantitatively confirmed in Table 1: large L1_2_ precipitates contain 70.15 at.% Ni, 5.45 at.% Cr, 6.22 at.% Fe, 5.38 at.% Al, and 12.85 at.% Ti, while fine L1_2_ precipitates formed during aging consist of 62.17 at.% Ni, 11.73 at.% Cr, 12.74 at.% Fe, 5.44 at.% Al, and 7.92 at.% Ti. In comparison, the bulk matrix has 41.07 at.% Ni, 25.91 at.% Cr, 27.34 at.% Fe, 3.08 at.% Al, and 2.78 at.% Ti. These results substantiate that Ni, Al, and Ti exhibit a strong tendency to partition into the precipitates, whereas Cr and Fe are largely retained in the matrix. Such pronounced elemental partitioning is a key factor contributing to the mechanical performance of the alloy. The Ni-Al-Ti-enriched L1_2_ precipitates act as strong barriers to dislocation movement, significantly impeding plastic deformation, while this chemical heterogeneity enhances the interactions between dislocations and precipitate/matrix interfaces, promoting both strain hardening and ductility. Therefore, the combined structural and chemical heterogeneity, as shown by the mappings in Figure 23d–h and the compositional analysis in Table 1, fundamentally underpins the simultaneous improvement of strength and ductility in this alloy system.

**Figure 24:**
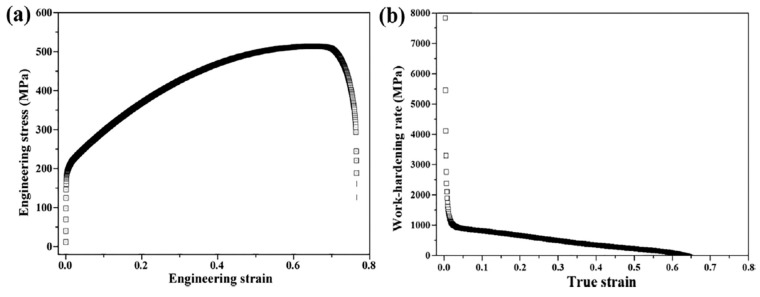
Tensile testing of $Ni_{50}Cr_{25}Fe_{25}$ MEA. (**a**): Engineering stress–strain curve. (**b**): Work-hardening rate–strain curve [17].

**Figure 25:**
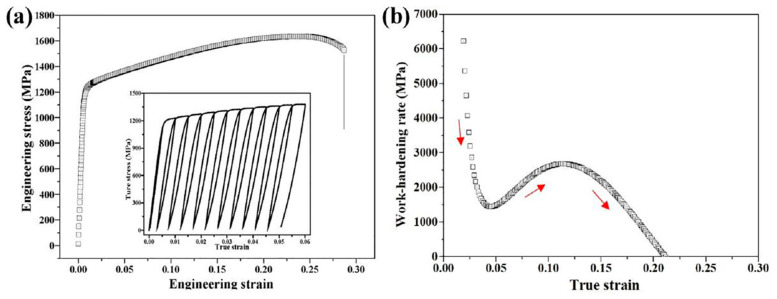
Tensile testing of $Ni_{46}Cr_{23}Fe_{23}Al_{4}Ti_{4}$ MEA. (**a**): Engineering stress–strain curve. (**b**): Work-hardening rate–strain curve [17].

Figure 24 and Figure 25 present the representative engineering stress–strain and work-hardening rate curves for the solid solution $Ni_{50}Cr_{25}Fe_{25}$ MEA and the $Ni_{46}Cr_{23}Fe_{23}Al_{4}Ti_{4}$ MEA, respectively. As shown in Figure 24, the $Ni_{50}Cr_{25}Fe_{25}$ alloy achieves superior ductility of ~78% but exhibits a relatively low yield strength (~187 MPa) and ultimate tensile strength (~520 MPa), exemplifying the typical strength–ductility trade-off. By contrast, Figure 25 illustrates the mechanical response of the DHS Al_4_Ti_4_ MEA, which combines outstanding strength and ductility, presenting a high yield strength of ~1203 MPa, an ultimate tensile strength of ~1633 MPa, and a notable elongation of ~28.7%. The inset in Figure 25a shows the loading–unloading–reloading test, further demonstrating pronounced HDI hardening.

When comparing the work-hardening rate curves, it is evident that the solid solution MEA exhibits a conventional two-stage decreasing trend, whereas the DHS Al_4_Ti_4_ MEA displays an additional increase in work-hardening rate within the true strain range of 4.5–11.4%. This unique stage reflects the activation of a distinct strengthening mechanism—HDI hardening—arising from the accumulation and propagation of GNDs at heterointerfaces (NGs/MGs, UFGs/MGs) caused by plastic strain incompatibility in heterogeneous grains. Furthermore, the presence of both large and dense fine L1_2_ precipitates (with a measured total volume fraction of ~26.03%, including ~12.27% from annealing and ~13.76% from aging) enhances precipitation strengthening. The nanoscale L1_2_ precipitates not only hinder dislocation mobility but can also serve as dislocation sources during deformation, significantly increasing the overall dislocation density and work-hardening response.

For the present MEA system, the theoretical yield strength ($\sigma_{yield}$) arises from the combined effects of lattice friction ($\sigma_{0}$), solid solution hardening ($\Delta \sigma_{s}$), HDI hardening ($\Delta \sigma_{HDI}$), and precipitation hardening ($\Delta \sigma_{p}$). This relationship can be mathematically expressed as follows:

(1) $$\begin{array}{c} \sigma_{yield}=\sigma_{0}+\Delta \sigma_{s}+\Delta \sigma_{HDI}+\Delta \sigma_{p} \end{array}$$

where $\sigma_{0}$ is the base lattice friction stress, taken as 187 MPa based on the tensile testing of the $Ni_{50}Cr_{25}Fe_{25}$ MEA. For the $Ni_{46}Cr_{23}Fe_{23}Al_{4}Ti_{4}$ MEA, due to the relatively low content of Al and Ti, the mechanical matrix is reasonably treated as $Ni_{50}Cr_{25}Fe_{25}$, with Al and Ti considered as solutes. The contribution from solid solution hardening is estimated using

(2) $$\begin{array}{c} \Delta \sigma_{s}=MG_{M}\frac{\varepsilon_{S}^{3/2}c^{1/2}}{700} \end{array}$$

where *M* is the Taylor factor, $G_{M}$ is the shear modulus, $\varepsilon_{s}$ is the solute misfit parameter, and c is the atomic concentration of solute. Our calculation gives $\Delta \sigma_{s}$ = 52.8 MPa. HDI hardening, reflecting the strengthening caused by the accumulation and propagation of geometrically necessary dislocations at heterointerfaces, is given by

(3) $$\begin{array}{c} \Delta \sigma_{HDI}=\frac{\sigma_{r}+\sigma_{u}}{2} \end{array}$$

where $\sigma_{r}$ and $\sigma_{u}$ are the reloading and unloading stresses obtained from LUR testing, respectively. In our measurements, $\Delta \sigma_{HDI}$ = 453 MPa. Precipitation hardening is expressed as

(4) $$\begin{array}{c} \Delta \sigma_{p}=\Delta \sigma_{p}^{A}+\Delta \sigma_{p}^{B} \end{array}$$

where the $\Delta \sigma_{p}^{A}$ results from large $L1_{2}$ precipitates, while $\Delta \sigma_{p}^{B}$ is attributed to the small $L1_{2}$ precipitates. Thus, based on the calculation, the precipitation hardening is $\Delta \sigma_{p}$~651.6 MPa. When all the effects are combined together, the calculated potential yield strength is ~1346.8 MPa which reasonably shows the agreement with the experimental one (~1203 MPa). It is worth mentioning that the primary strength improvement is contributed from the HDI and precipitation hardening, but the precipitation hardening provides more, approximately 50% of strength.

While precipitation hardening is a widely used and effective method for enhancing the strength of metallic alloys, its practical application in industry is often accompanied by several notable challenges. Firstly, the process can increase manufacturing costs due to the necessity for precise heat treatment protocols, tightly controlled temperature management, and the use of high-quality raw materials [59]. Furthermore, the risk of component distortion during heat treatment becomes significant, particularly when dealing with large or complex components, which can complicate the production process and affect dimensional accuracy [60]. Another important drawback is the phenomenon of over-aging, whereby excessive or prolonged heat treatment causes the precipitates to coarsen, resulting in a decline in the intended strengthening effect and potentially reducing the material’s overall performance [61]. In addition, the hardening response in precipitation-hardened alloys is highly sensitive to both the initial composition of the alloy and the specific parameters used in post-processing. Small deviations in alloy composition or inconsistencies in the heat treatment process can lead to considerable differences in the kinetics and distribution of precipitation, ultimately resulting in variability in mechanical properties within and between production batches [62].

These practical limitations can restrict the broader industrial implementation of precipitation-hardened alloys, especially in applications where reliability and consistency are critical. Therefore, careful optimization of alloy design, heat treatment protocols, and process monitoring is essential to minimize these drawbacks and ensure stable and reproducible material performance [63].

## 5. Proposed Approach Based on Gradient Structure and Multi-Material SLM

### 5.1. Research Status

With the rapid advancement of SLM technology, the fabrication of multi-material components—especially those incorporating dissimilar metals such as 18Ni300, 316L stainless steel, and CuSn10—has become a key research focus in advanced manufacturing. However, combining different metallic powders in a single process introduces significant scientific and engineering challenges that can directly impact microstructural quality and mechanical properties.

One major challenge lies in the thermal mismatch between dissimilar alloys, which is rooted in differences in thermal expansion coefficients and melting points. Barath Kumar and Manikandan systematically assessed how severe thermal gradients generated during SLM can produce high residual stresses at material interfaces, leading to risks such as part distortion, microcracking, or even interfacial delamination [64]. Brennan further analyzed various defect formation mechanisms in metal additive manufacturing and emphasized that residual stress plays a critical role in the reliability of multi-material structures [65]. Interfacial diffusion and elemental mixing also pose significant obstacles. Cui and co-workers demonstrated that rapid thermal cycling during SLM of steel and aluminum alloys can cause intense interdiffusion at interfaces, with the formation of brittle intermetallic compounds and local chemical inhomogeneities [66]. Wu’s group investigated Ti and Al multi-material systems and revealed that uncontrolled interfacial reactions can severely undermine mechanical integrity [67]. Similarly, Miao and colleagues studied the bonding of 316L with AlSi10Mg, finding that element diffusion and interface products can greatly reduce the ductility and strength of the composite parts [68]. Residual stress accumulation is complicated by the distinctive heat input and thermal conductivity of each material type. Huang demonstrated that high residual stress arising from mismatched thermal behavior may surpass the interfacial bonding strength, resulting in delamination or crack propagation during or even after fabrication [69]. The interface-affected zone (IAZ), representing microstructural and compositional transitions between two materials, is particularly sensitive to SLM process parameters. Zhang’s work clearly showed that the thickness and continuity of the IAZ play a crucial role in determining both the interfacial strength and yield behavior of 18Ni300/316L heterogeneous structures [70].

To address these challenges, Ma explored process parameter optimization to tailor the microstructure and mechanical properties of SLM-fabricated 18Ni300 [71]. Ferreira’s team compared both conventionally produced and SLM-manufactured 18Ni300, concluding that careful control of post-processing treatments is necessary to mitigate adverse interface effects [72]. In recent years, researchers have placed increasing emphasis on in situ monitoring and finite element simulations to predict and alleviate thermal and mechanical mismatches during multi-material additive manufacturing.

In summary, while multi-material SLM offers promising possibilities for fabricating functionally graded and heterogeneous structures, it remains essential to address challenges associated with thermal mismatch, interfacial diffusion, residual stress accumulation, and delamination risks. Continuous advances in interface engineering and process optimization are crucial for realizing reliable and high-performance multi-material additively manufactured components.

### 5.2. Solutions

Drawing inspiration from natural gradient structures and multiple strengthening mechanisms, an approach is proposed that combines gradient structuring with heterogeneous material selection. Subsequent post-processing treatments—including hetero-deformation-induced hardening and various heat treatments—can be used to enhance the material’s strength to an ultrahigh level while maintaining ductility.

**Figure 26:**
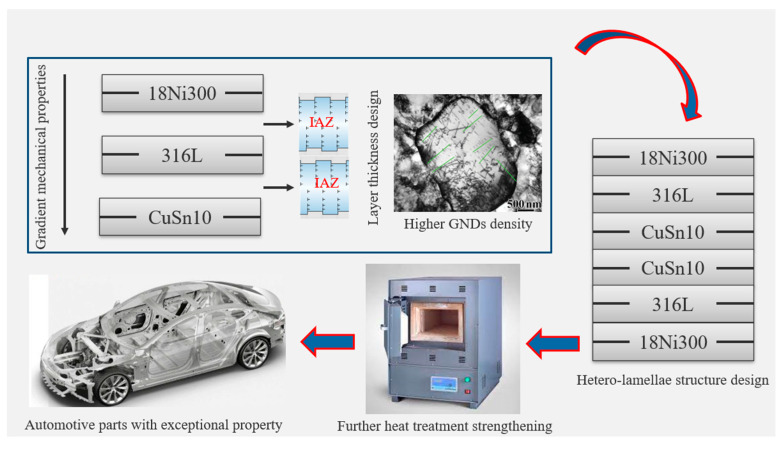
The strengthening mechanism of the new idea.

First, three types of materials (18Ni300, 316L, CuSn10) can be obtained from the manufacturer with powder formation. Then, according to current additive manufacturing processes, SLM technology is selected to fabricate the bulk material to form the gradient structure. In the gradient structure, the GNDs strengthening mechanism can be firstly applied to enhance the strength. Furthermore, according to the IAZ, each layer thickness can be optimized for SLM process to maximize the forward- and back-stress to further improve the material strength/ductility, referred to in Section 4.1, which is due to the accumulation of the GNDs. Additionally, further optimization through symmetrical structure design and the application of heat treatments (such as annealing and aging) can implement precipitation strengthening mechanisms, as discussed in Section 4.2. This combination is expected to result in significant strength improvement while preserving desirable mechanical properties. The designed material is satisfied and can be applied for manufacturing the automotive parts with exceptional properties. The strengthening mechanism and the detailed structural layout of the new idea are shown in Figure 26.

## 6. Conclusions

This study systematically explores natural gradient structures present in organisms such as lobster exoskeletons and bamboo, with the aim of inspiring the design of advanced automotive materials exhibiting exceptional mechanical performance. By integrating HDI hardening and precipitation hardening mechanisms, the proposed approach effectively overcomes the traditional strength–ductility trade-off. Additive manufacturing, particularly SLM, was successfully employed to fabricate bulk gradient structures, and subsequent microstructural analyses confirmed that both optimized interface-affected zones and controlled precipitation distributions play vital roles in property enhancement. The results demonstrate that by mimicking gradient hierarchical architectures from biology and combining them with tailored post-processing, it is possible to achieve ultrahigh strength while maintaining high ductility and significant energy absorption capacity.

Looking forward, several challenges remain for the large-scale manufacturing and practical implementation of such bioinspired architectures. These include ensuring the uniformity and reproducibility of gradient structures during mass production, developing cost-effective and scalable fabrication processes, and addressing potential hurdles in quality control. Furthermore, this research opens up opportunities for application-specific customization. For instance, by tuning architecture and processing techniques, these materials can be optimized for use as crash energy absorbers, battery enclosures, or other critical components requiring superior mechanical robustness in automotive and other engineering fields. Alignment with sustainability goals is another important aspect. The developed strategy supports the use of recyclable materials and additive manufacturing processes that may reduce material waste. Future studies should include comprehensive life cycle assessments (LCA) to evaluate the environmental impact and recyclability of these advanced materials, ensuring their contribution to sustainable development.

In summary, this work not only advances the state of the art in bioinspired material design but also sets the stage for practical, scalable, and sustainable applications in next-generation automotive engineering and beyond. Specifically, the main conclusions of this study are as follows:

1. Inspired by biological gradient structures such as lobster exoskeletons and bamboo, this work proposes an advanced material design strategy for automotive applications. The integration of HDI hardening and precipitation hardening effectively enhances both strength and ductility, overcoming the traditional strength–ductility trade-off.
2. The study demonstrates that bioinspired gradient-structured materials, combined with tailored post-processing, can achieve ultrahigh strength, excellent ductility, and superior energy absorption, meeting the lightweight and performance requirements of modern automotive engineering.

## Figures and Tables

**Table 1:** Chemical constitution of precipitates phases of $Ni_{46}Cr_{23}Fe_{23}Al_{4}Ti_{4}$ MEA [17].

Phases	Compositions (at.%)				
	Ni	Cr	Fe	Al	Ti
$\mathrm{Large}\;L1_{2}$	70.15	5.45	6.22	5.38	12.85
$\mathrm{Fine}\;L1_{2}$	62.17	11.73	12.74	5.44	7.92
Bulk material	41.07	25.91	27.34	3.08	2.78

## Data Availability

No new data were created or analyzed in this study. Data sharing is not applicable to this article.
